# Specific Upregulation of a Cotton Phytoene Synthase Gene Produces Golden Cottonseeds with Enhanced Provitamin A

**DOI:** 10.1038/s41598-018-19866-1

**Published:** 2018-01-22

**Authors:** Dan Yao, Yi Wang, Qian Li, Xufen Ouyang, Yaohua Li, Chuannan Wang, Lingli Ding, Lei Hou, Ming Luo, Yuehua Xiao

**Affiliations:** Biotechnology Research Center, Chongqing Key Laboratory of Application and Safety Control of Genetically Modified Crops, Southwest University, Beibei, Chongqing, 400715 China

## Abstract

Provitamin A (PVA) bio-fortification of crops offers a sustainable strategy to prevent the prevalence of vitamin A deficiency (VAD), one of the world’s major public health problems. The present work aimed to enhance PVA accumulation in cottonseed, the main by-product in the production of cotton fibers and the third largest source of edible plant oil in the world. On the basis of comprehensive identification of carotenoid synthase genes and their expression levels in various cotton tissues, we selected phytoene synthase as the target for manipulating carotenoid biosynthesis in the developing cottonseeds. After functional verification in transgenic tobacco, a cotton phytoene synthase gene (*GhPSY2D*) driven by a seed-specific promoter was transformed into cotton. The transgenic cottonseeds showed golden appearance and contained over 6-fold higher carotenoid contents in the extracted oil than the non-transgenic control. Thin layer chromatograph analysis indicated that the main PVA carotenoid β-carotene was predominant in the transgenic cottonseeds, but undetectable in the wild-type control. By simultaneously providing economically valuable fibers and edible oils, the transgenic cottons bio-fortified with β-carotene in seeds may be a new powerful tool against VAD in low-income regions.

## Introduction

Vitamin A (retinol) is an essential nutrient for vertebrates, including humans. Vitamin A deficiency (VAD) may result in a series of disorders in animals, including impaired growth, reproduction, epithelial integrity, and disease resistance^1^. In human, this nutrient deficiency causes xerophthalmia, including night blindness, and increases the risk of infant morbidity and mortality from measles and diarrhoea in children^2^. VAD has been one of major human health problems for a long time. Although the overall prevalence of worldwide VAD has significantly decreased in the last two decades, this deficiency is still unacceptably serious in some regions, such as south Asia and sub-Sahara Africa^3,4^. This may be largely attributed to insufficient dietary diversification, unsuccessful food fortification, and the restricted vitamin A capsule delivery in these regions^3^. Compared to the supply of vitamin A capsule and vitamin A-rich animal-source foods, growing and consuming crops with high PVA level (PVA biofortified crops) is more sustainable and effective to alleviate VAD prevalence, especially in the low-income regions^5,6^. Several PVA biofortified crops have been reported to prevent the VAD prevalence effectively in various populations^7,8^.

PVA include a group of carotenoids containing at least one non-substituted β-ring, such as β-carotene, α-carotene, and β-cryptoxanthin, which are synthesized via a complex carotenoid pathway (Supplementary Fig. S1)^6,9–11^. In higher plants, carotenoids are synthesized in the plastids from geranylgeranyl pyrophosphate (GGPP), which is the precursor for multiple pathways and contains four molecules of isopentenyl pyrophosphate originating from glyceraldehyde phosphate and pyruvate (Supplementary Fig. S1). Phytoene synthase (PSY) catalyzes the first committed reaction of carotenoid pathway to synthesize phytoene from two GGPP molecules. Accumulating evidence indicates that PSY is the key regulatory enzyme in the biosynthesis of carotenoids^6,11–13^. Biotechnological strategies have been successfully employed to increase PVA level (especially β-carotene) in a wealth of crops, resulting in golden canola^14^, rice^15–17^, wheat^18^, sorghum^19^, corn^20,21^, cassava^22^, potato^23^, sweet potato^24^, tomato^25^, soybean^26^ and banana^5^. These works mainly adopted two strategies, i.e. to up-regulate PSY solely^5,14^ or with other synthases^5,15–20^ to promote carotenoid biosynthesis, and to express an *Orange* gene to enhance carotenoid accumulation^15,21,24,27^. Recently, it was reported that the Orange protein might promote carotenoid biosynthesis via stabilizing the key synthase PSY^28,29^.

Cotton (*Gossypium*) is the leading natural fiber crop in the world, and is one of the major economic drivers in developing countries. In addition to fiber, cottonseed is an important source of edible oil (ranking 3rd in the world) and high-quality proteins^30,31^. Therefore, cotton is a potential supporting crop for poor regions to improve the economic and nutrient status simultaneously. PVA bio-fortified cotton and cottonseed oil may be a powerful tool against VAD prevalence in low-income cotton-growing regions, for example in south Asia and sub-Sahara Africa^3,32,33^. Our work aimed to increase PVA content of cottonseed and the resultant cottonseed oil. To this end, a functional PSY gene predominantly expressed in cotton was cloned, and upregulated specifically in the developing seeds. The transgenic cottons, with normal growth and development, produced golden cottonseeds and cottonseed oil fortified with β-carotene. This work significantly improved the nutritional value of cottonseeds, which provided this cash crop with the potential to prevent VAD prevalence.

## Results

### Identification and expression analysis of carotenoid synthase genes in *Gossypium*

**Figure 1:**
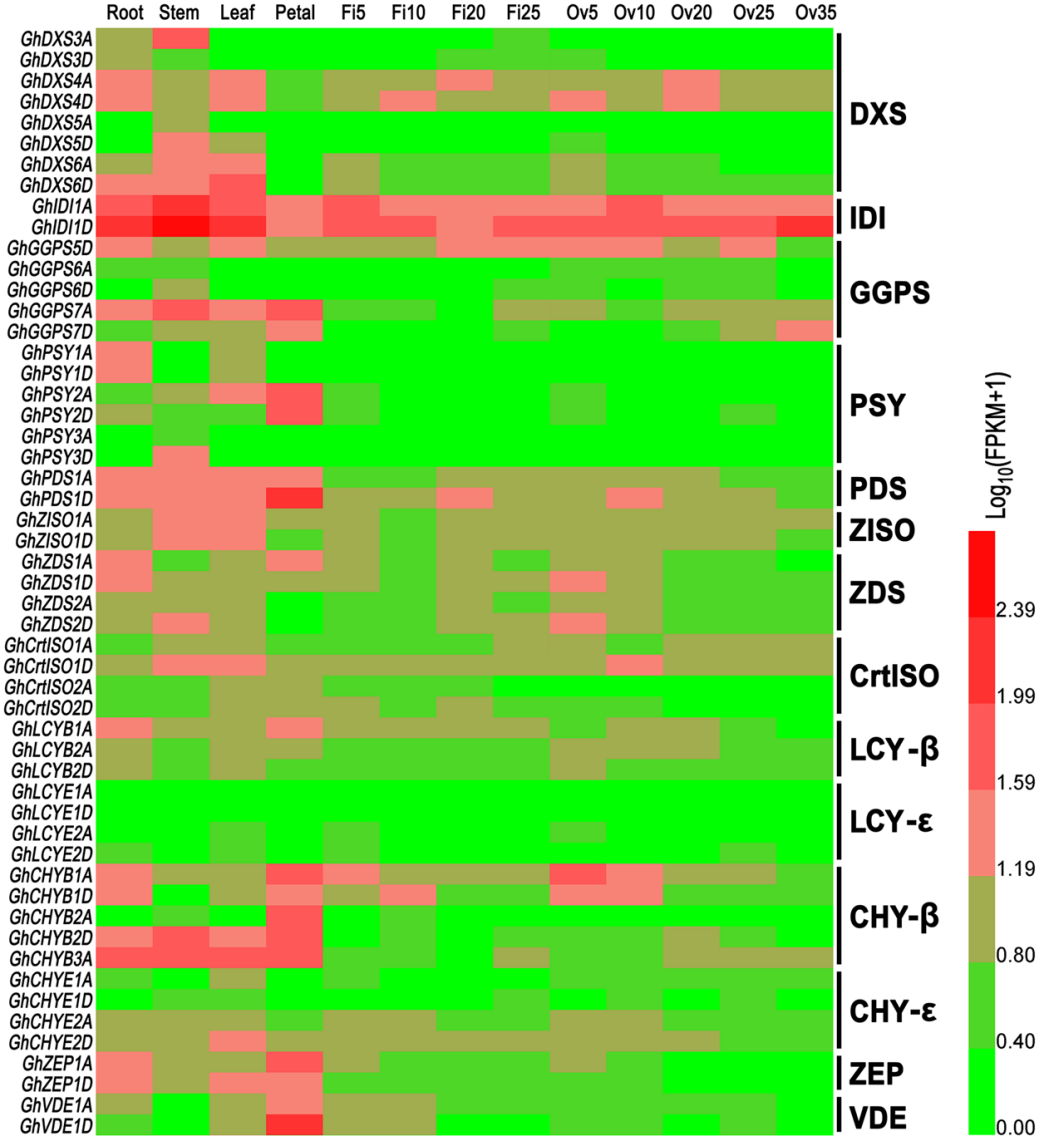
Transcript levels of carotenoid synthase genes in various upland cotton tissues. The gene expression levels (FPKM) in root, stem, leaf, petal, and fibers (Fi5–25) and ovules (Ov5–35) of various days post anthesis (DPA) are converted to Log_10_(FPKM + 1) and illustrated as seven classes in the heat map. Genes are named as the abbreviation of species and enzyme plus code plus A or D to indicate the subgenome origin. Enzymes are abbreviated as in Supplementary Fig. S1. Transcript levels are inferred from the transcriptomic data^34^. The genes with very low transcript level (total FPKM < 2) are omitted.

To characterize intrinsic carotenoid biosynthesis in various cotton tissues, especially in the developing seeds, we compared transcript levels of carotenoid related genes using transcriptomic data^34^. Firstly, we identified 36, 29 and 54 carotenoid synthase genes from the assembled cotton genomes of *G*. *raimondii*, *G*. *arboreum* and *G*. *hirsutum*, respectively (Table 1)^34–36^. These genes encoded all the enzymes catalyzing violaxanthin synthesis from GGPP and 3 key enzymes upstream to GGPP. As shown in Fig. 1, the transcript levels of the investigated carotenoid synthase genes varied with tissues and development stages, indicating that the carotenoid biosynthesis in cotton was developmentally regulated at transcription level. Generally, the transcript levels of carotenoid synthase genes were lower in the developing fibers and ovules compared to those in the roots, stems, leaves and petals. In the developing ovules, the PSY and LYC-ε genes had only a trace of transcription, while genes coding the rest carotenoid synthases had low-to-moderate expression. The transcript profile of carotenoid synthase genes suggested that the transcription of PSY genes may be a limiting factor of carotenoid biosynthesis in cottonseeds.

**Table 1:** The coding genes of carotenoid synthases identified in cottons. Enzymes are abbreviated as in Supplementary Fig. S1. Genes are named alphabetically according to their IDs in *G*. *raimondii*, and homeologs annotated in different genomes (D5, A2, Dt1 and At1) are list in the same line.

Enzymes	Genes	*G*. *raimondii* (D5)	*G*. *arboreum* (A2)	*G*. *hirsutum*-Dt1	*G*. *hirsutum*-At1
DXS	*GoDXS1*	*Gorai*.*004G030400*	*Cotton_A_06764*	*—*	*—*
	*GoDXS2*	*Gorai*.*004G030800*	*Cotton_A_06768*	*—*	*—*
	*GoDXS3*	*Gorai*.*004G030900*	*Cotton_A_06769*	*Gh_D08G0270*	*Gh_A08G0193*
	*GoDXS4*	*Gorai*.*004G111100*	*Cotton_A_21777*	*Gh_D08G1005*	*Gh_A08G2461*
	*GoDXS5*	*Gorai*.*008G093600*	*Cotton_A_31168*	*Gh_D12G2793*	*Gh_A12G0784*
	*GoDXS6*	*Gorai*.*011G184200*	*Cotton_A_39687*	*Gh_D10G1640*	*Gh_A10G2292*
IDI	*GoIDI1*	*Gorai*.*004G262800*	*Cotton_A_05787*	*Gh_D08G2391*	*Gh_A08G1997*
GGPS	*GoGGPS1*	*Gorai*.*002G251300*	*Cotton_A_00433*	*Gh_D01G2110*	*Gh_A01G1866*
	*GoGGPS2*	*Gorai*.*002G251400*	*—*	*—*	*—*
	*GoGGPS3*	*Gorai*.*005G054800*	*—*	*—*	*—*
	*GoGGPS4*	*Gorai*.*005G120800*	*—*	*—*	*—*
	*GoGGPS5*	*Gorai*.*007G051300*	*Cotton_A_02023*	*Gh_D11G0475*	*—*
	*GoGGPS6*	*Gorai*.*007G206700*	*Cotton_A_32196*	*Gh_D11G1883*	*Gh_A11G1725*
	*GoGGPS7*	*Gorai*.*011G102700*	*Cotton_A_17013*	*Gh_D10G0907*	*Gh_A10G0844*
	*GoGGPS8*	*Gorai*.*011G102800*	*—*	*—*	*—*
	*GoGGPS9*	*Gorai*.*011G103000*	*—*	*—*	*—*
	*GoGGPS10*	*Gorai*.*011G133100*	*—*	*—*	*—*
PSY	*GoPSY1*	*Gorai*.*001G083700*	*Cotton_A_12202*	*Gh_D07G0746*	*Gh_A07G0668*
	*GoPSY2*	*Gorai*.*006G009400*	*Cotton_A_14731*	*Gh_D09G0078*	*Gh_A09G0080*
	*GoPSY3*	*Gorai*.*010G126900*	*Cotton_A_36110*	*Gh_D06G1167*	*Gh_A06G0932*
	*GoPSY4*	*Gorai*.*012G039100*	*Cotton_A_29856*	*Gh_D04G0308*	*Gh_A05G3296*
PDS	*GoPDS1*	*Gorai*.*011G149900*	*Cotton_A_36228*	*Gh_D10G1327*	*Gh_A10G1167*
ZISO	*GoZISO1*	*Gorai*.*005G080900*	*Cotton_A_34612*	*Gh_D02G0721*	*Gh_A02G0673*
ZDS	*GoZDS1*	*Gorai*.*007G284900*	*Cotton_A_14988*	*Gh_D11G2616*	*Gh_A11G2306*
	*GoZDS2*	*Gorai*.*013G201600*	*Cotton_A_12871*	*Gh_D13G1826*	*Gh_A13G1497*
CrtISO	*GoCrtISO1*	*Gorai*.*002G153800*	*Cotton_A_34115*	*Gh_D01G1197*	*Gh_A01G1122*
	*GoCrtISO2*	*Gorai*.*002G218000*	*Cotton_A_27012*	*Gh_D01G1808*	*Gh_A01G1557*
LCY-β	*GoLCYB1*	*Gorai*.*006G113300*	*Cotton_A_26267*	*Gh_D09G0946*	*Gh_A09G0916*
	*GoLCYB2*	*Gorai*.*013G002400*	*Cotton_A_20533*	*Gh_D13G0026*	*Gh_A13G0010*
LCY-ε	*GoLCYE1*	*Gorai*.*001G012900*	*Cotton_A_05719*	*Gh_D07G2366*	*Gh_A07G0117*
	*GoLCYE2*	*Gorai*.*009G032900*	*Cotton_A_09351*	*Gh_D05G0313*	*Gh_A05G0229*
CHY-β	*GoCHYB1*	*Gorai*.*006G199400*	*Cotton_A_27296*	*Gh_D09G1728*	*Gh_A09G1634*
	*GoCHYB2*	*Gorai*.*008G249600*	*Cotton_A_32712*	*Gh_D12G2438*	*Gh_A12G2678*
	*GoCHYB3*	*Gorai*.*008G274800*	*Cotton_A_01286*	*—*	*Gh_A12G2302*
CHY-ε	*GoCHYE1*	*Gorai*.*007G372200*	*Cotton_A_05513*	*Gh_D11G3263*	*Gh_A11G2878*
	*GoCHYE2*	*Gorai*.*013G180200*	*Cotton_A_26341*	*Gh_D13G1644*	*Gh_A13G1336*
ZEP	*GoZEP1*	*Gorai*.*007G213500*	*Cotton_A_24363*	*Gh_D11G3469*	*Gh_A11G1774*
VDE	*GoVDE1*	*Gorai*.*007G268500*	*Cotton_A_18131*	*Gh_D11G2473*	*Gh_A11G2173*

### Cloning and functional analysis of the *GhPSY2* gene

To manipulate carotenoid biosynthesis in developing cottonseeds, we firstly cloned the coding sequences of the predominantly-expressed cotton *PSY2* genes (Fig. 1) and analyzed their biological functions in transgenic tobacco. The coding regions of *GhPSY2A* and *GhPSY2D* were amplified from the leaf cDNA of upland cotton line T586, and their sequences were identical to those in the assembled TM-1 genome. Both *GhPSY2A* and *GhPSY2D* encoded proteins of 398 aa. GhPSY2A/2D were highly similar to plant group I PSYs, and distantly related to group II and III (Supplementary Fig. S2A). Multiple sequence alignment indicated that both GhPSY2 proteins had conserved DxxxD motifs, substrate binding pocket, catalytic residues and active site lid motifs (Supplementary Fig. S2B). Along with the expression data, these results suggested that *GhPSY2A/2D* might encode biologically functional PSYs.

**Figure 2:**
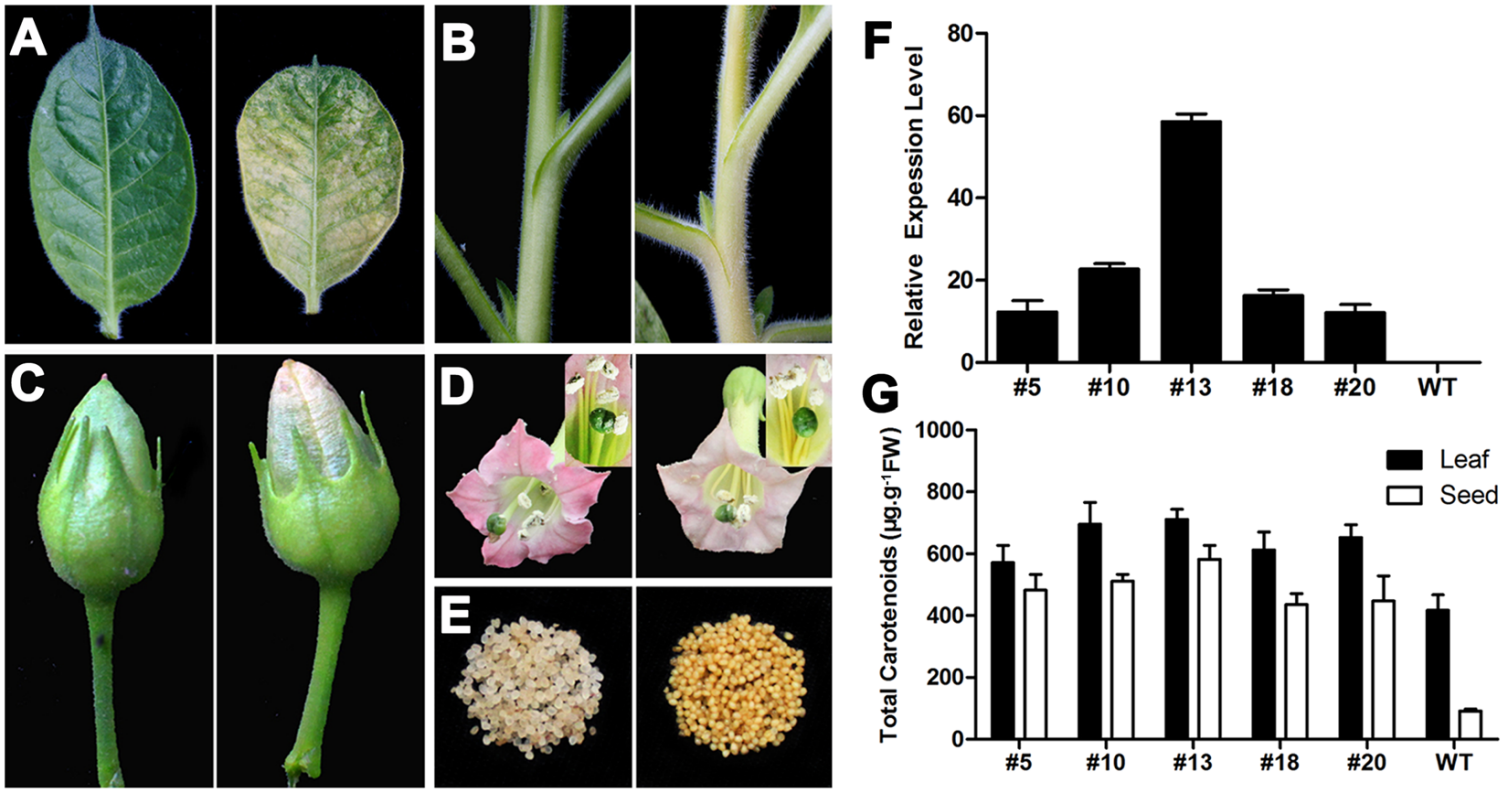
Overexpression of *GhPSY2D* promotes carotenoid biosynthesis in tobacco. (**A**–**E**) Phenotypic comparison of *GhPSY2D* over-expresser (transformant #13, right) and the wild-type control (a null segregant of transformant #13, left); (**A**) Leaves; (**B**) Stems; (**C**) Capsules; (**D**) Flower and stamens; (**E**), 12-DPA seeds; (**F**) Transcript levels of *GhPSY2D* in wild-type control (WT) and *GhPSY2D* over-expressers (#5, #10, #13, #18 and #20); (**G**) Carotenoid quantification in leaves and 12-DPA seeds. Error bars indicate standard deviations of 3 biological replicates.

To further elucidate the biological functions of the cloned PSY genes, *GhPSY2D* was overexpressed in tobaccos. Compared with the wild-type control, the *GhPSY2D* over-expressers accumulated higher levels of carotenoids, and had golden appearance in several organs, such as leaves, stems, filaments, and developing seeds (Fig. 2A-E). Meanwhile, the over-expressers showed retarded growth and dwarf phenotype (Supplementary Fig. S3) as reported in tomatoes^37^. These results indicated that *GhPSY2D* was biologically functional to promote carotenoid synthesis in plants.

### Golden cottonseed from specific upregulation of *GhPSY2D*

**Figure 3:**
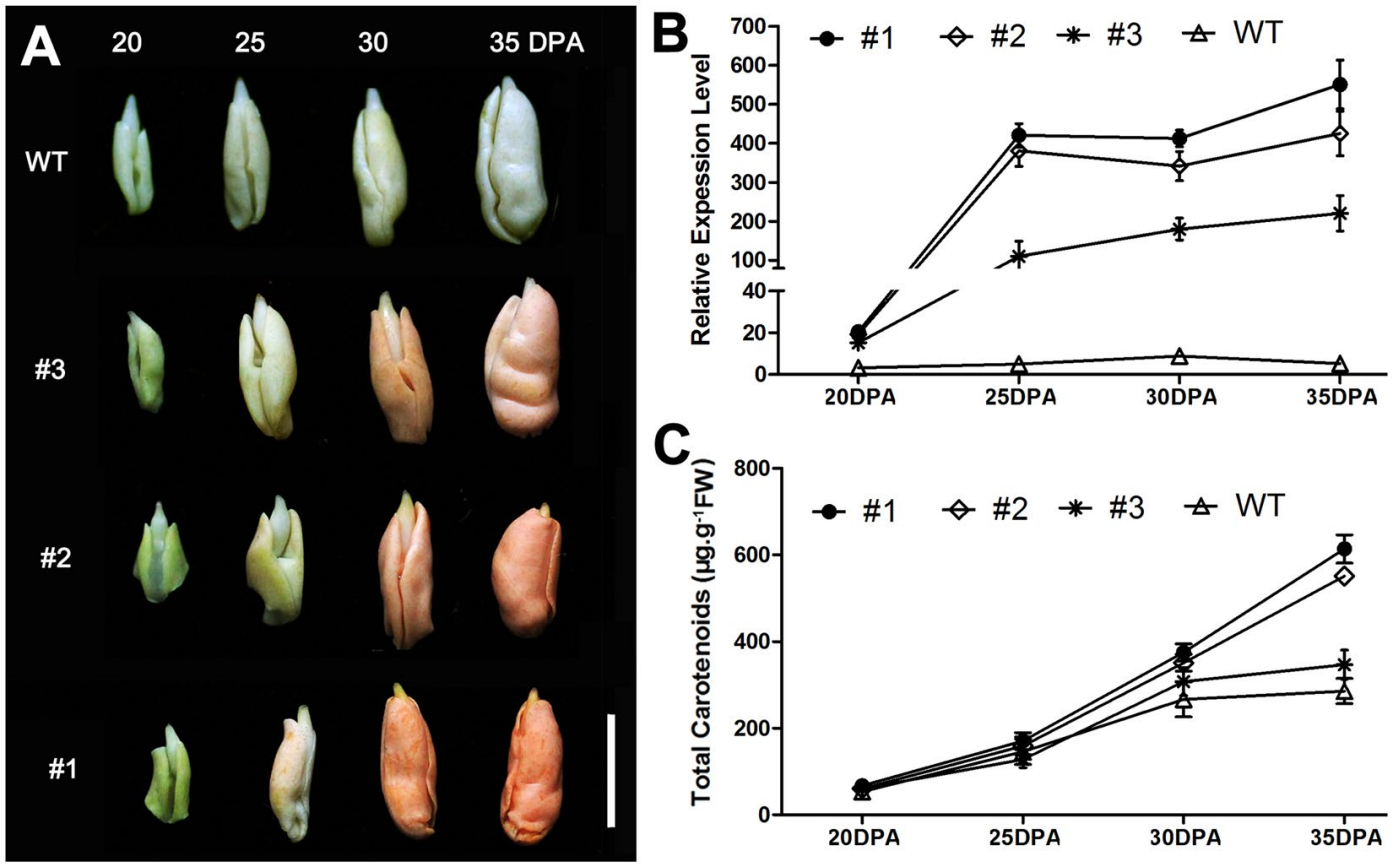
*GhPSY2D* expression and carotenoid accumulation in the developing cottonseeds. Colors (**A**), *GhPSY2D* transcript levels (**B**) and carotenoid contents (**C**) in the developing embryos (20–35 DPA) of the transformants #1, #2 and #3, and the wild-type control (WT, a null segregant of transformant #1) are indicated.

**Figure 4:**
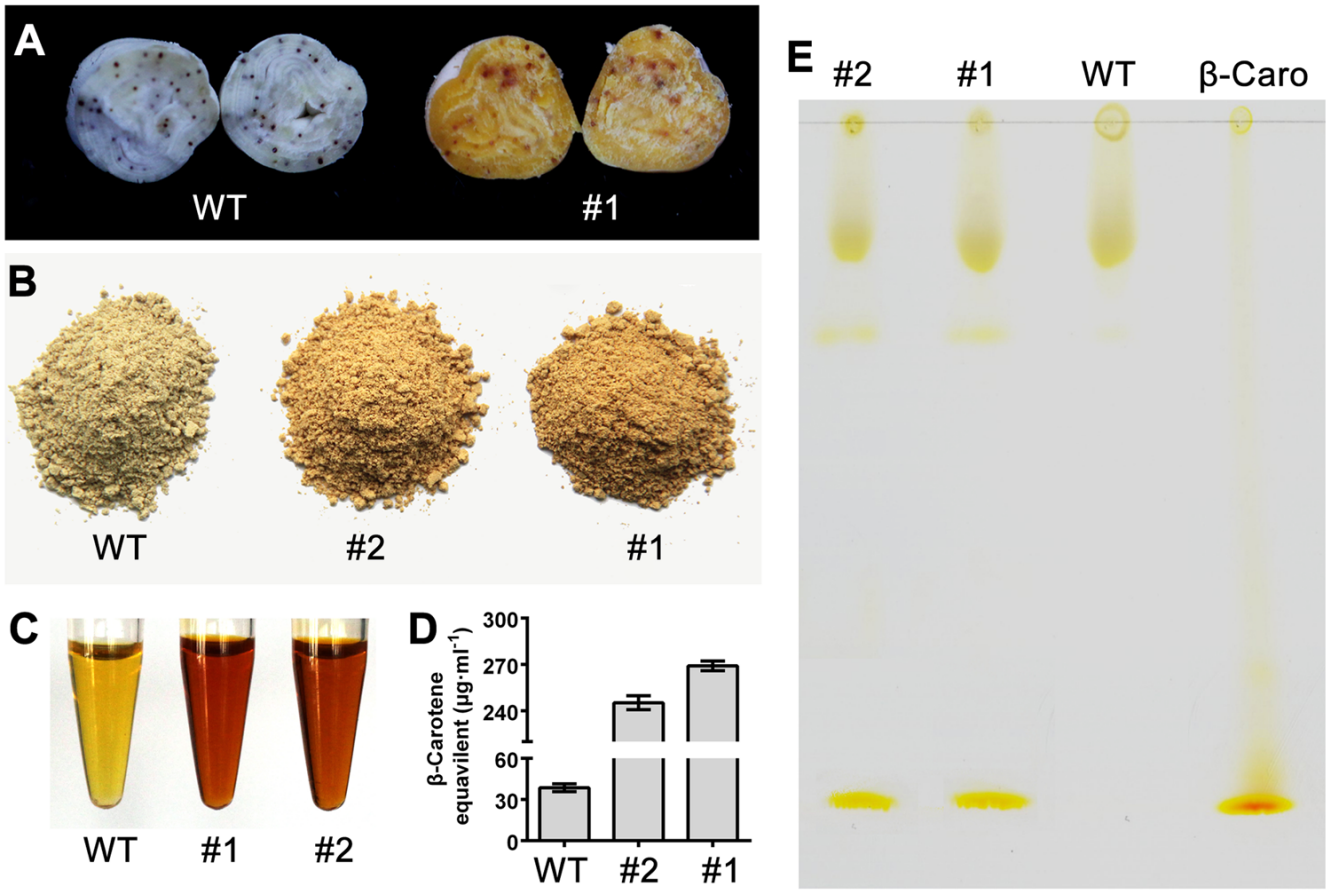
Carotenoids in mature cottonseeds. (**A**) Transverse view of mature seed kernels; (**B**) Seed kernel powder; (**C**) Cottonseed oils; (**D**) Total carotenoid content in cottonseed oils; (**E**) TLC analysis of carotenoid components in cottonseed oils. Standards β-carotene is separated along with samples.

To increase the carotenoid content of cottonseeds, *GhPSY2D* was constructed downstream to a seed-specific promoter *pV*^38,39^, and transformed into upland cotton. We finally obtained 3 transformants (#1, #2 and #3) with *GhPSY2D* highly expressed in developing seeds (Fig. 3). As expected, *GhPSY2D* transcript (Fig. 3B) and carotenoid (Fig. 3C) levels in the transgenic embryos dramatically increased at the mid-to-late stage (after 25 DPA) and the embryos turned golden accordingly (Fig. 3A). The mature *pV:GhPSY2D* cottonseeds had golden kernel and the carotenoid contents in the extracted oil of transgenic cottonseeds were significantly higher (over 6-fold) than that from the wild type (Fig. 4A–D). Importantly, thin layer chromatography (TLC) analysis indicated that the major carotenoid increased in the transgenic cottonseeds was β-carotene (Fig. 4E), which was the main plant PVA carotenoid. Moreover, the *pV:GhPSY2D* cottons showed no obvious defect in plant growth and fiber development compared to the wild-type control (Supplementary Fig. S4), although the germination rates of transgenic cottonseeds decreased as reported in Arabidopsis (Supplementary Fig. S5)^40^. These results demonstrated that the transgenic cottonseeds were successfully bio-fortified for PVA, rendering the transgenic cotton a potential tool against VAD in cotton-growing regions including south Asia and sub-Sahara Africa^32,33^.

## Discussion

The objective of the present work is to generate PVA bio-fortified cottonseeds, which may be useful tool against VAD prevalence in low-income regions. On the basis of comprehensive identification of carotenoid synthase genes in the assembled cotton genomes, we compared the transcript levels of these genes in various cotton tissues, including developing ovules, and demonstrated that the transcription of PSY genes might be the limiting factor for carotenoid biosynthesis and accumulation in cottonseeds. Next, we cloned the *GhPSY2* genes, and confirmed their biological functions by over-expression in tobacco. Finally, the *GhPSY2D* gene driven by a seed-specific promoter *pV* was transformed into upland cotton, and significantly expressed in the mid-to-late embryos. The resultant transgenic cottonseeds had golden kernel, and the extracted oil contained significantly higher level of carotenoids, especially β-carotene (the major active PVA). Given cottons are widely grown as cash crop in south Asia and sub-Sahara Africa^32,33^, the PVA bio-fortified transgenic cottonseeds may be quite potential to prevent VAD prevalence in these regions.

To prevent VAD prevalence, a wealth of crops were bred or engineered to contain high levels of carotenoids or PVAs^6,24^. Compared with the previously reported PVA bio-fortified crops, cotton has significant advantages in the battle against VAD prevalence. Firstly, cottonseeds are lipid-rich and PVAs are easily extracted with cottonseed oil (Fig. 4)^30,31,41^. As reported, fat in diet significantly increase the β-carotene bioavailability^42,43^, implying that enhanced PVA in cottonseed and cottonseed oil should be more easily utilized by human compared with these in starch-rich crops. Secondly, human malnutrition, including VAD, generally occurs in developing regions, where the demands on economic development and nutrient improvement, somewhat mutually dependent and contradictory, are both pressing. As one of the most important cash crops in developing regions including south Asia and sub-Sahara Africa^32,33^, cotton plays a crucial role in economic development and poverty reduction. PVA bio-fortified transgenic cottons can simultaneously meet the demands on PVA supply and income increments, therefore may become a powerful tool in the battle against poverty and VAD prevalence. Notably, our method is easy to combine with other tactics, such as specific inhibition of gossypol synthesis via RNA interfering^31^, to further enhance the nutrient value and utilization of cottonseeds.

Carotenoid biosynthesis in plants involves a multi-step complex pathway (Fig. 1)^6,9–11^. The final carotenoid level and profile in a certain tissue are collectively determined by the substrate accessibility and enzyme activities catalyzing these synthesis steps. For example, the golden rice from upregulation of PSY and CrtI mainly accumulates β-carotene, instead of lycopene, attributing to the constitutively expressed intrinsic carotenoid synthases^44^. Before the sequenced genomes and comprehensive expression data available, designing strategies to engineer carotenoid and other secondary metabolites largely depended on experience and generally multiple enzymes were simultaneously targeted, which substantially added difficulty in gene manipulation^5,15,26,45^. In this work, we designed the transgenic strategy for PVA bio-fortification on the basis of comprehensive identification and expression analysis of carotenoid synthase genes using assembled genomes and transcriptomic data (Table 1 and Fig. 1), and obtained the golden cottonseed by specifically upregulating a single gene (*GhPSY2D*). Consistent with the very low transcript levels of the encoding genes of LCY-ε, ZEP and VDE, and moderate expressions of PDS, ZISO, ZDS, crtISO, LCY-β and CHY-β genes in the late-stage cottonseeds (35 DPA, Fig. 1), *GhPSY2D* upregulation in cottonseeds mainly promoted the accumulation of β-carotene and another carotenoid, probably zeaxathin (Fig. 4E). These results indicated that comprehensive evaluation of all the genes involved in a certain pathway may be a useful foundation for manipulation of secondary metabolites in plants.

## Methods

### Identification and expression analysis of carotenoid synthase genes in cotton

To identify the carotenoid synthase genes in cottons, Arabidopsis proteins were employed as probe to query homologous sequences from *G*. *raimondii* genome in phytozome (https://phytozome.jgi.doe.gov/)^46^. The resultant *G*. *raimondii* sequences were aligned with all the annotated proteins of certain synthase, and subjected to construct a neighbor-joining tree with 1000 replicates of bootstrap test in MEGA6.0^47^. The homologs clustered with certain Arabidopsis carotenoid synthase were regarded as carotenoid synthases in *G*. *raimondii*. The corresponding orthologous genes in *G*. *arboreum* and *G*. *hirsutum* were identified with a standalone BLAST software using *G*. *raimondii* genes as probe^48^.

The transcript levels of all predicted *G*. *hirsutum* genes were evaluated routinely using public available transcriptomic data^34^ released by Zhang’s lab (http://mascotton.njau.edu.cn). The transcript levels (in FPKM, fragment per kilobase per million) of identified carotenoid synthase genes in various *G*. *hirsutum* tissues were depicted as heat map using the program HemI 1.0^49^.

### RNA Extraction and qRT-PCR

Total RNAs were extracted from approximately 100 mg of plant tissues using a rapid plant RNA extraction kit (Aidlab, Beijing, China). The first-stranded cDNAs were synthesized from 1 µg total RNA using a reverse transcriptase kit (TaKaRa, Dalian, China). Quantitative PCR was performed on a CFX96 real-time PCR detection system using SYBR Green Supermix (Bio-Rad, CA, USA) and gene-specific primers (Table S1). The thermocycling parameters were as follows: 95 °C for 3 min, followed by 40 cycles of 95 °C for 5 s, 57 °C for 20 s, and a standard melting curve to monitor PCR specificity. The actin^50^ and histone3 (AF024716)^51^ gene were amplified as internal control in tobacco and cotton, respectively. The analyses included three biological replicates and data were analyzed using the software Bio-Rad CFX Manager 2.0 provided by the manufacturer.

### Cloning and bioinformatics analysis of *GhPSY2*

A pair of primers (GhPSY2-U and GhPSY2-D, Supplementary Table S2) encompassing the full-length ORF, designed according to the *GhPSY2* sequences identified in *G*. *hirsutum*, was employed to amplify the cDNA coding sequences from leaves. The reaction included 1 μl first-stranded cDNA, 0.2 μM each primer, and 1 × PrimeSTAR max Premix (TaKaRa, Dalian, China), and amplified for 35 cycles of 98 °C for 10 s, 56 °C for 15 s and 72 °C for 40 s. The PCR products were cloned into pGEM-T easy vector (Promega, Shanghai, China), sequenced in BGI (Shenzhen, China), and further compared to the *GhPSY2* genes identified in the assembled *G*. *hirsutum* genome. GhPSY2 proteins were aligned with Arabidopsis, rice and maize PSYs from Phytozome (https://phytozome.jgi.doe. gov/)^46^ using ClustalW, and the NJ tree was constructed with 1000 replicates of bootstrap test in MEGA6.0^47^.

### Vector construction and plant transformation

A modified pBI121 vector p5 (pBI121-GN), containing selection marker *NPTΙΙ* and *GUS* genes, was used to construct the plant expression vectors^52^. The cDNA sequences of *GhPSY2D* ORF was excised from the cloning vector pGEM-T easy using *Bam*HΙ and *Eco*RΙ, and inserted downstream to a CaMV35S promoter in the p5 vector restricted by the same enzymes, resulting in the overexpression vector. To construct seed-specific expression vectors, the promoter of *Phaseolus vulgaris* β-type phaseolin storage protein gene (*pV*, GenBank accession no. J01263.1)^39^, were amplified with restriction sites of *Hin*dΙΙΙ and *Bam*HΙ (Table S1). The promoter was constructed upstream to *GhPSY2D* by replacing the CaMV35S promoter in the overexpression vector using *Hin*dΙΙΙ and *Bam*HΙ sites. All these expression vectors were transferred into *Agrobacterium tumefaciens* strains (LBA4404), and the resulting *Agrobacterium* strain was used for tobacco and cotton (Jimian No. 14) transformation as previously described^50,52^.

### Carotenoid extraction and analysis

Carotenoids in fresh tobacco tissues and developing cottonseeds were extracted according Fuentes’ method^53^ with some modification. In brief, approximately 500 mg of fresh tissues were ground to fine powder in liquid nitrogen, extracted for 15 min in 3 ml hexane/acetone/ethanol (2:1:1 v/v/v) with shacking. Two to three successive extractions were performed to remove carotenoids until the tissues were colorless. The extracts were combined, dried with nitrogen, and re-suspended in 1 ml acetone. Total carotenoids were measured spectrophotometrically at 474 nm, and quantified according to a standard curve of β-carotene. The extractions were repeated in three biological replicates.

Carotenoids in dry mature cottonseeds were extracted along with oil with Soxhlet extractor (Buchi B-811, Switzerland) with ether. The resultant cottonseed oil were 20-fold diluted in acetone and subjected to spectrophotometric quantification of total carotenoids as mentioned above and TLC analysis^54^. Ten microliter of diluted cottonseed oil were loaded and separated on Silica gel plate (0.2 mm-thick, Jiangyou Silica Gel Co., Yantai, Shangdong, China) along with 5 μg β-carotene standards. The plate was developed in hexane:ether:acetone (60:30:20, v/v) and photographed directly.

## Electronic supplementary material

Supplementary Information
