# Supplementary material for: Specific Upregulation of a Cotton Phytoene Synthase Gene Produces Golden Cottonseeds with Enhanced Provitamin A
Source: Sci Rep. 2018 Jan 22;8:1348. doi: 10.1038/s41598-018-19866-1 (PMC5778082; doi:10.1038/s41598-018-19866-1)
Supplement: Supplementary file 1 — Supplementary Information [file 41598_2018_19866_MOESM1_ESM.doc]

**Supplementary Information**

Specific Upregulation of a Cotton Phytoene Synthase Gene Produces Golden Cottonseeds with Enhanced Provitamin A

Dan Yao^+^, Yi Wang^+^, Qian Li, Xufen Ouyang, Yaohua Li, Chuannan Wang, Lingli Ding, Lei Hou, Ming Luo, Yuehua Xiao*


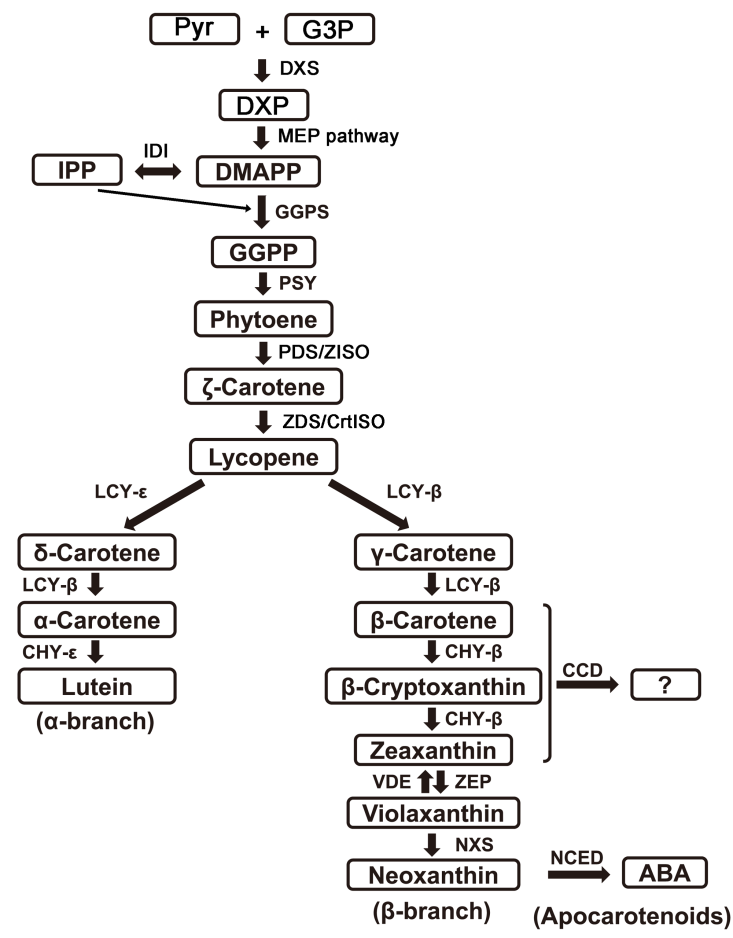


**Supplementary Figure S1.** Carotenoid biosynthesis pathway and related enzymes in plants (Modified from Kang *et al*, 2017). DXS, 1-deoxy-D-xylulose 5-phosphate synthase; IDI, isopentenyl pyrophosphate:dimethylallyl pyrophosphate isomerasee; GGPS, geranylgeranyl pyrophosphate synthase; PSY, phytoene synthase; PDS, phytoene desaturase; ZISO, 15-cis-ζ-carotene isomerase; ZDS, ζ-carotene desaturase; CrtISO, prolycopene isomerase; LCY-β, lycopene β-cyclase; LCY-ε, lycopene ε-cyclase; CHY-ε, ε-ring hydroxylase; CHY-β, β-carotene hydroxylase; ZEP, zeaxanthin epoxidase; VDE, violaxanthin de-epoxidase; NXS, neoxanthin synthase; CCD, carotenoid cleavage dioxygenase; NCED, 9-cis-epoxycarotenoids dioxygenase; Pyr, pyruvate; G3P, glyceraldehyde 3-phosphate; DXP, 1-deoxy-D-xylulose 5-phosphate; IPP, isopentenyl pyrophosphate; DMAPP, dimethylallyl pyrophosphate; GGPP, geranylgeranyl pyrophosphate; ABA, abscisic acid.


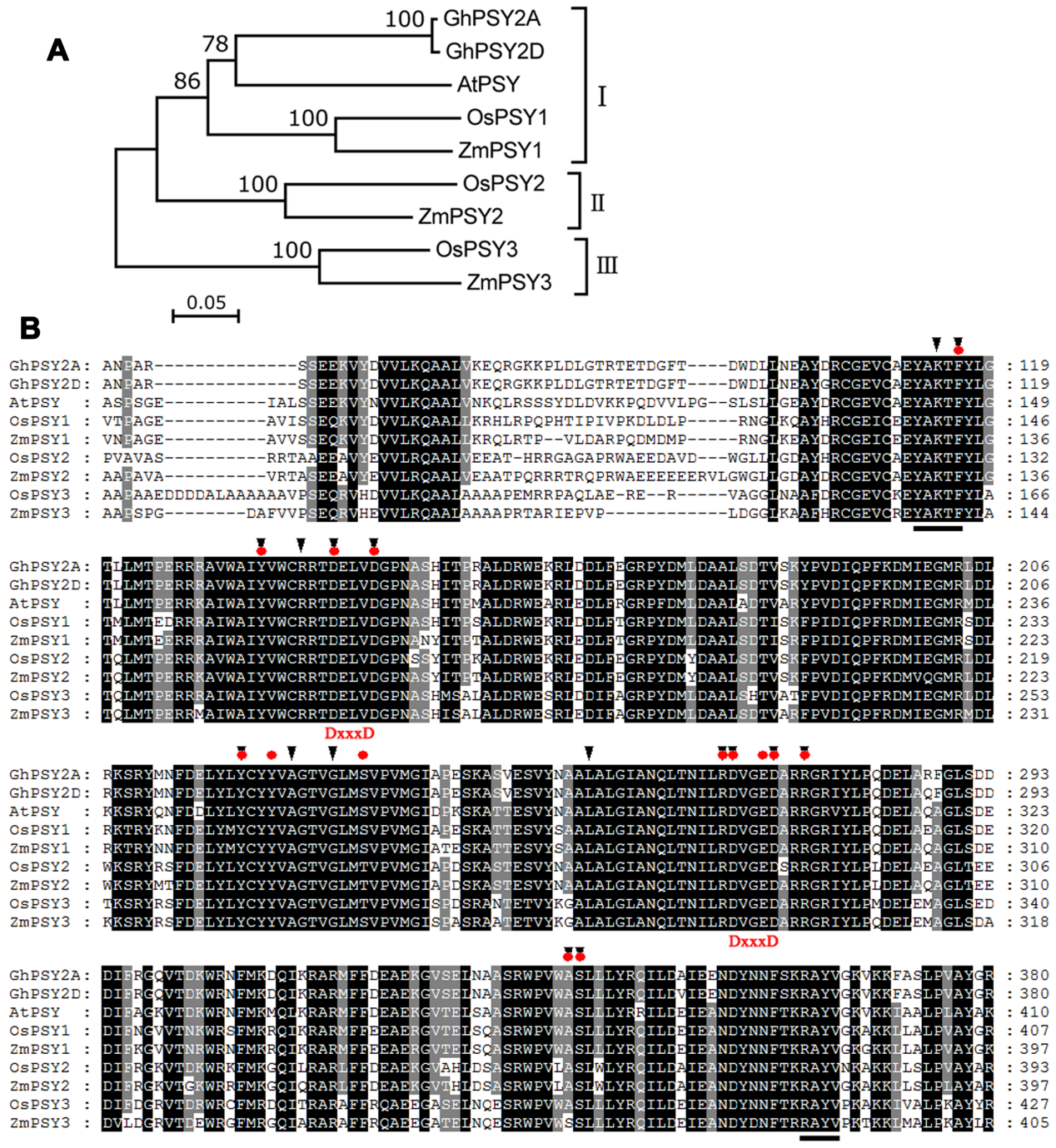


**Supplementary Figure S2.** Phylogenetic analysis of GhPSY2A/2D and representive plant PSYs. A, The phylogenetic tree. The PSY sequences are from Arabidopsis (AtPSY, AAA32836), rice (OsPSY1, AAS18307; OsPSY2, AAK07735; OsPSY3, DQ356431), and maize (ZmPSY1, P49085; ZmPSY2, AAQ91837; ZmPSY3, DQ356430). Amino acid sequences are aligned using Clustal W and a neighbor-joining tree is constructed with a 1000-replicate bootstrap test. The scale bar indicates the estimated number of amino acid replacements per site. B, Multiple sequence alignment. Aspartate rich motifs (DxxxD) are shown in red. Black arrowheads direct the amino acid residues composing of the substrate binding pocket. Red circles mark the catalytic residues. Black bars indicate active site lid motifs.


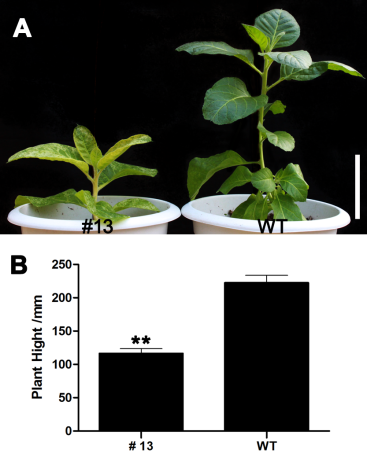


**Supplementary Figure S3.** Dwarfism phenotype of *GhPSY2D*-overexpressing tobacco. A, Forty-d plants of transformant #13 and its null segregant (WT). Bar = 10cm. B, The heights of T1 plants of transformant #13 and its null segregant (WT). Error bars indicated SEM of 6 plants. Asterisks indicate significant differences (p ˂ 0.01) relative to the wild-type control.


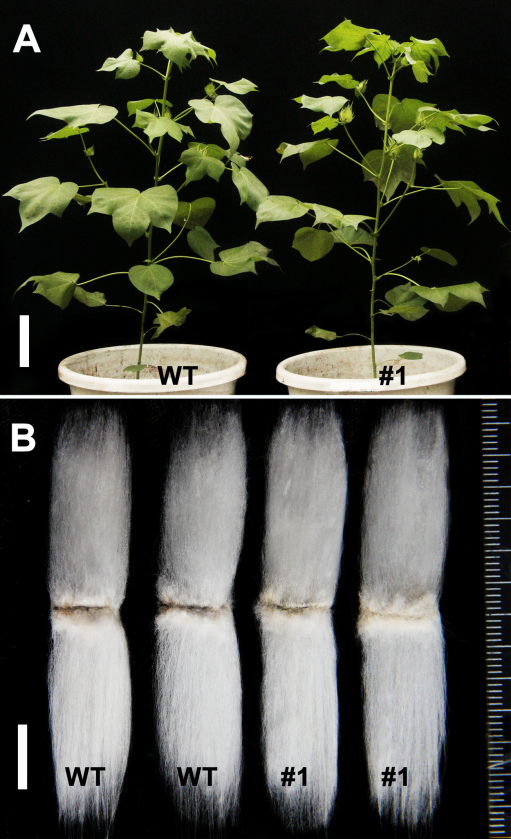


**Supplementary Figure S4.** Plant (A) and mature fiber (B) of *pV:PSY2* cotton（#1）and the wild-type control (WT, null segregant of transformant #1). Plants are photograghed 60d after germination. The bar in A = 10 cm and that in B = 1 cm.


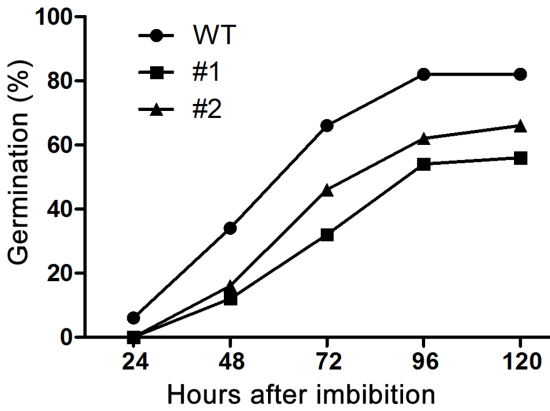


**Supplementary Figure S5.** The germination of *pV:GhPSY2D* cottonseeds. Fifty dry seeds of wild-type control and *pV:GhPSY2D* transformants #1 and #2 were spread on wetted toilet paper. The germinated seeds were counted after incubation at 32℃ for 24h, 48h, 72h, 96h and 120h.

| Primer name | Primer sequence (5'-3') | Usage |
| --- | --- | --- |
| GhPSY2-U | **GGATCC**ATGGCTGGTGTTCTTCTTTGG | Upstream primer to amplify *GhPSY2* ORFs |
| GhPSY2-D | **GAATTC**AGAACAATTTGGAAGTGCC | Downstream primer to amplify *GhPSY2* ORFs |
| pV-U | **AAGCTT**GTACTCCCAGTATCATTATAG | Upstream primer to amplify the *pV* promoter |
| pV-D | **GGATCC**AGTAGTATTGAATATGAGTTGGG | Downstream primer to amplify the *pV* promoter |
| GhPSY2D-F | GCCGCATTAGCCCTCGGAATT | Upstream primer for qRT-PCR analysis of *GhPSY2D* |
| GhPSY2D-R | CATCGTCCGACAGTCCGAACT | Downstream primer for qRT-PCR analysis of *GhPSY2D* |
| NtACT-F | ATGCCCTCCCACATGCTATT | Upstream primer to amplify the internal actin gene for the qRT-PCR analysis in tobacco |
| NtACT-R | AACATGGTAGAGCCACTG | Downstream primer to amplify the internal actin gene for the qRT-PCR analysis in tobacco |
| GhHIS3-F | AAATCCATGGGTTTCCGC | Upstream primer to amplify the internal histone3 gene for the qRT-PCR analysis in cotton |
| GhHIS3-R | CTACCACTACCATCATGGC | Downstream primer to amplify the internal histone3 gene for the qRT-PCR analysis in cotton |

Supplementary Table S1. Sequences and usage of primers used in this study
